# Cancer survival disparities worsening by socio-economic disadvantage over the last 3 decades in new South Wales, Australia

**DOI:** 10.1186/s12889-017-4692-y

**Published:** 2017-09-14

**Authors:** Hanna E. Tervonen, Sanchia Aranda, David Roder, Hui You, Richard Walton, Stephen Morrell, Deborah Baker, David C. Currow

**Affiliations:** 1 School of Health Sciences, Centre for Population Health Research, University of South Australia, GPO Box 2471, Adelaide, SA 5001 Australia; 2 Cancer Institute NSW, GPO Box 41, Alexandria, Sydney, NSW 1435 Australia; 3 Cancer Council Australia, GPO Box 4708, Sydney, NSW 2001 Australia; 4 School of Public Health and Community Medicine, University of New South Wales, UNSW, Sydney, 2052 Australia

**Keywords:** Neoplasms, Australia, Socioeconomic factors, Rural population, Cultural diversity, Survival analysis

## Abstract

**Background:**

Public concerns are commonly expressed about widening health gaps. This cohort study examines variations and trends in cancer survival by socio-economic disadvantage, geographical remoteness and country of birth in an Australian population over a 30-year period.

**Methods:**

Data for cases diagnosed in New South Wales (NSW) in 1980–2008 (*n* = 651,245) were extracted from the population-based NSW Cancer Registry. Competing risk regression models, using the Fine & Gray method, were used for comparative analyses to estimate sub-hazard ratios (SHR) with 95% confidence intervals (CI) among people diagnosed with cancer.

**Results:**

Increased risk of cancer death was associated with living in the most socio-economically disadvantaged areas compared with the least disadvantaged areas (SHR 1.15, 95% CI 1.13–1.17), and in outer regional/remote areas compared with major cities (SHR 1.05, 95% CI 1.03–1.06). People born outside Australia had a similar or lower risk of cancer death than Australian-born (SHR 0.99, 95% CI 0.98–1.01 and SHR 0.91, 95% CI 0.90–0.92 for people born in other English and non-English speaking countries, respectively). An increasing comparative risk of cancer death was observed over time when comparing the most with the least socio-economically disadvantaged areas (SHR 1.07, 95% CI 1.04–1.10 for 1980–1989; SHR 1.14, 95% CI 1.12–1.17 for 1990–1999; and SHR 1.24, 95% CI 1.21–1.27 for 2000–2008; *p* < 0.001 for interaction between disadvantage quintile and year of diagnosis).

**Conclusions:**

There is a widening gap in comparative risk of cancer death by level of socio-economic disadvantage that warrants a policy response and further examination of reasons behind these disparities.

## Background

Socio-economic differences in cancer survival have been reported in many countries [1–6], including in Australia for cancers overall [7, 8] and specific cancer sites [7, 9, 10]. However, it has been unclear whether survival disparities are narrowing or widening [3, 5, 10–14]. In the United Kingdom (UK), socio-economic cancer survival inequalities persisted or widened over 1986–2001 [13]. By 2006 in the UK, the deprivation gap in 1-year survival had narrowed slightly with survival inequalities remaining wide [3]. A Japanese study reported overall improving cancer survival without significant widening of inequalities over 1993–2004 [5], whereas a study from New Zealand found an increase in cancer survival disparities across income groups over 1991–2004 [14]. A recent Australian study reported that socio-economic cancer survival disparities have remained for several cancer sites over 1996–2008, despite overall increases in cancer survival [10]. These examples indicate a need to reliably identify existing and changing cancer survival disparities to inform health-service provision.

In Australia, apart from people of low socio-economic status, potentially disadvantaged population groups include residents of remote/rural areas and people born overseas [15]. Australians living in remote and rural areas generally have poorer cancer survival than people living in metropolitan areas [11, 16, 17]. A recent Australian study reported a widening gap by residential remoteness in breast cancer survival over 1987–2007 [11]. Australia has a large immigrant population comprising 28% of the population (6.6 million in 2014) [18]. Data for 1981–2007 indicated that migrants in Australia generally have lower cancer mortality than the Australian-born although there were differences between migrant groups [19]. Large-scale, whole-population studies examining cancer survival differences between migrant groups in Australia are lacking.

Various methods have been used to examine cancer survival disparities, however, the impact of competing risk events has been rarely acknowledged. Competing risk regression modelling takes directly into account the effects of competing risk events which are censored/ignored in cause-specific survival models [20]. If these competing events co-vary with the outcome or factors of interest, not accounting for competing events could lead to biased estimates of risk [21]. Acknowledging competing risk events is especially important when studying older people with multiple co-morbidities and higher mortality rates from other causes during long follow-up periods, such as for people with cancer [22]. One Australian study reported cancer patients having 50% higher risk of death due to non-cancer causes compared with the general population [23].

Results of competing risk regression may be especially relevant for guiding resource allocation in the presence of multiple competing risk events [20, 21]. The present study examines variations and trends in cancer survival by socio-economic disadvantage, geographical remoteness and country of birth in New South Wales (NSW) over almost three decades (1980–2008), taking into account competing causes of death.

## Methods

### Study design, setting and data sources

NSW is the most populous state in Australia with an estimated resident population of seven million people in 2008 (33% of the Australian population) [24]. Population-based New South Wales Cancer Registry (NSW CR) data were used for this study. Cases of primary invasive cancer diagnosed in NSW residents have been reported to the NSW CR since 1972 (excluding non-melanoma skin cancers). All notifications relating to a particular cancer are linked to a single person and if the same person is diagnosed with another primary cancer in a different site, that cancer counts as a second case. NSW CR data include demographic information, cancer diagnosis and death data, and residential address at time of diagnosis. The NSW Registry of Births, Deaths and Marriages, the Australian Bureau of Statistics (ABS) and the National Death Index (NDI) provide death data including deaths due to cancer and from other (non-specified) causes. Death processing is very complete in NSW and cause of death information is well ascertained in the NSW CR [25].

This cohort study included cancer cases diagnosed between January 1980 and December 2008, apart from: [1] those with central nervous or lymphohaematopoietic tumours, excluded due to high proportions of missing values or non-applicable summary stage information (*n* = 76,912); [2] those with only death certificate information available (DCO cases) and those cancers found only at post-mortem (*n* = 12,006); and [3] those with missing information on socio-economic disadvantage (*n* = 2171).

Approval for the study was obtained from the NSW Population and Health Services Research Ethics Committee (NSW PHSREC 2012 07410). Data analysed for this paper are not able to be shared on any publicly available repository due to NSW privacy laws.

### Measures

#### Study variables

Residential remoteness was indicated by remoteness areas (RAs) based on the Accessibility/Remoteness Index of Australia (ARIA+) and categorised as major cities (reference category), inner regional and outer regional/remote areas [26]. ARIA+ indices are derived from measures of physical road distance between populated localities and the nearest urban centre. RA categories were determined by aggregating remoteness allocations of corresponding Census Collection Districts (CDs) for cases diagnosed in 2000–2008. For cases diagnosed in 1980–1999, Statistical Local Area (SLA) population based correspondence was used to calculate RAs [27].

The Australian Bureau of Statistics (ABS) has created four Socio-Economic Indexes for Areas (SEIFAs) [28]. This study utilized the Index of Relative Socio-Economic Disadvantage (IRSD) which summarises information about the socio-economic conditions of people and households in a specified area. For this study, the IRSD was based on CD of residence at the time of diagnosis using the CD boundaries of the most recent ABS Census. CDs were the smallest geographic units with SEIFA information available. In the 2006 Census, they included approximately 250 households in urban areas and far fewer households in rural areas [28]. Socio-economic disadvantage was categorised into equal-population quintiles (1: least disadvantaged (reference category) - 5: most disadvantaged).

For the purposes of this study, country of birth was categorised into Australia (reference category), other predominantly English speaking, predominantly non-English speaking countries and unknown (no country-of-birth information available from the NSW CR). Other English speaking countries include New Zealand, the United Kingdom, Ireland, the United States of America, Canada, and South Africa, based on the ABS categorisation [29].

#### Variables for adjustment

Age in years at the time of cancer diagnosis was categorised as <65, 65–79 and ≥80 years for descriptive analyses, and as 0–39, 40–49, 50–59, 60–64, 65–69, 70–74, 75–79, 80–84 and 85+ years in regression models.

The NSW CR is the only Australian cancer registry that has recorded summary stage (extent of disease) for all solid malignant tumours since its inception [30]. Summary stage is defined as the highest degree of spread based on all diagnostic and therapeutic information available to the NSW CR up to four months after the cancer diagnosis, in accordance with international guidelines used by cancer registries worldwide [30]. Summary stage was categorised as localised, regional, distant or unknown.

Cancer site classification was according to the International Classification of Diseases Oncology (ICD-O-3) [31]. Following an ICD-classification change to ICD-O-3 in 2003, data for earlier years were re-mapped from the previous ICD-9 classification, and recoded where necessary, to comply with ICD-O-3 standards. Cases with central nervous or lymphohaematopoietic tumours were excluded (C70-C72, M959-M973, M976, M980-M994, M9963, M9987, C42 or C77 with M974, M975, M995-M996 (excluding M9963)). Remaining cancer sites were classified as follows: stomach (C16), colon/rectum (C18,C19-C21), liver (C22), pancreas (C25), lung (C33,C34), melanoma of the skin (C44 with M872-M879), breast (C50), cervix (C53), uterus (C54,C55), prostate (C61), kidney (C64-C66,C68), bladder (C67), ill-defined, unspecified & rare sites (C26,C39,C42,C48,C76,C80), and remaining malignant cancer sites (‘other cancers’).

Other variables for adjustment included sex and diagnostic period (1980–1989, 1990–1999 and 2000–2008).

### Statistical analyses

Competing risk regression models using the Fine & Gray approach were used to examine hazard of death due to cancer [32] using Stata *stcrreg* command [33]. Competing risk regression models the subhazard function of an event of interest in the presence of competing events (also known as the cumulative incidence function). In this study, we were interested in cumulative incidence of the cancer death in the presence of other causes of deaths. Death from the incident cancer was the outcome of interest, and deaths from other causes were regarded as competing events. Cases were followed from the date of cancer diagnosis to death or to 1 January 2009, which ever occurred first.

Multivariable models included the study variables (remoteness, socio-economic disadvantage quintile and country of birth), and were adjusted for sex, age and diagnostic period (Model 1); with further adjustment for cancer site (Model 2); and for summary stage (Model 3). Trends over time were examined using interaction terms and period-stratified analyses. Wald’s chi-square test was used to examine the overall significance of interactions between categorical study variables and year of diagnosis, expressed as a continuous variable. Cancer site-specific analyses were conducted for lung, colon/rectum, breast, prostate, melanoma, stomach, cervix and liver cancers. These sites were selected because they represent high-burden cancers and/or cancers considered likely to be affected by social disparities [2, 6–8].

Results were presented as sub-hazard ratios (SHRs) with 95% confidence intervals (CIs). Proportional hazards assumptions were examined using -ln[−ln(survival)] curves. We also conducted sensitivity analyses excluding cases with unknown summary stage and cases with unknown country of birth. All analyses were performed using Stata Statistical Software: Release 14 (College Station, TX: StataCorp LP, 2015).

## Results

A total of 651,245 cases with a mean follow-up of 5.5 years were included in the study. Of these, 358,011 (55.0%) were males, 281,795 (43.3%) were aged <65 years at diagnosis, and 281,783 (43.3%) were diagnosed with a localised cancer (Table 1). Most people lived in major cities (*n* = 458,455, 70.4%) and were born in Australia (*n* = 444,879, 68.3%). Fewer people lived in the least socio-economically disadvantaged areas than in the most disadvantaged areas (*n* = 115,352, 17.7% in quintile 1 vs. *n* = 144,534, 22.2% in quintile 5).

**Table 1:** Characteristics of the study population overall and by remoteness, socio-economic disadvantage and country of birth category, NSW 1980–2008

	All	Remoteness			SEIFA quintile					Country of birth			
		Major cities	Inner regional	Outer regional/Remote	1	2	3	4	5	Australia	Other English	Non-English	Unknown
	*(n = 651,245)*	*(n = 458,455)*	*(n = 141,831)*	*(n = 50,959)*	*(n = 115,352)*	*(n = 118,022)*	*(n = 130,111)*	*(n = 143,226)*	*(n = 144,534)*	*(n = 444,879)*	*(n = 68,944)*	*(n = 93,042)*	*(n = 44,380)*
	n (%)^b^	n (%)^b^	n (%)^b^	n (%)^b^	n (%)^b^	n (%)^b^	n (%)^b^	n (%)^b^	n (%)^b^	n (%)^b^	n (%)^b^	n (%)^b^	n (%)^b^
Sex													
Male	358,011 (55.0)	247,353 (54.0)	80,894 (57.0)	29,764 (58.4)	60,849 (52.8)	63,987 (54.2)	71,964 (55.3)	80,415 (56.2)	80,796 (55.9)	242,324 (54.5)	38,771 (56.2)	51,566 (55.4)	25,350 (57.1)
Female	293,234 (45.0)	211,102 (46.1)	60,937 (43.0)	21,195 (41.6)	54,503 (47.3)	54,035 (45.8)	58,147 (44.7)	62,811 (43.9)	63,738 (44.1)	202,555 (45.5)	30,173 (43.8)	41,476 (44.6)	19,030 (42.9)
Age^a^													
< 65	281,795 (43.3)	201,289 (43.9)	58,658 (41.4)	21,848 (42.9)	56,631 (49.1)	53,899 (45.7)	55,758 (42.9)	57,772 (40.3)	57,735 (40.0)	186,292 (41.9)	26,599 (38.6)	43,847 (47.1)	25,057 (56.5)
65–79	272,415 (41.8)	188,097 (41.0)	62,278 (43.9)	22,040 (43.3)	42,786 (37.1)	46,918 (39.8)	54,767 (42.1)	63,333 (44.2)	64,611 (44.7)	190,277 (42.8)	29,354 (42.6)	38,084 (40.9)	14,700 (33.2)
≥ 80	96,987 (14.9)	69,032 (15.1)	20,889 (14.7)	7066 (13.9)	15,923 (13.8)	17,197 (14.6)	19,582 (15.1)	22,112 (15.4)	22,173 (15.3)	68,308 (15.4)	12,990 (18.8)	11,110 (11.9)	4579 (10.3)
Summary stage													
Localised	281,783 (43.3)	200,462 (43.7)	60,520 (42.7)	20,801 (40.8)	56,004 (48.6)	53,486 (45.3)	56,010 (43.1)	59,226 (41.4)	57,057 (39.5)	190,129 (42.7)	27,706 (40.2)	36,426 (39.2)	27,522 (62.0)
Regional	135,648 (20.8)	97,486 (21.3)	27,858 (19.6)	10,304 (20.2)	24,823 (21.5)	24,529 (20.8)	26,788 (20.6)	29,332 (20.5)	30,176 (20.9)	95,211 (21.4)	15,094 (21.9)	22,190 (23.9)	3153 (7.1)
Distant	107,067 (16.4)	76,682 (16.7)	22,079 (15.6)	8306 (16.3)	16,109 (14.0)	18,464 (15.6)	21,406 (16.5)	24,635 (17.2)	26,453 (18.3)	75,315 (16.9)	12,852 (18.6)	17,453 (18.8)	1447 (3.3)
Unknown	126,747 (19.5)	83,825 (18.3)	31,374 (22.1)	11,548 (22.7)	18,416 (16.0)	21,543 (18.3)	25,907 (19.9)	30,033 (21.0)	30,848 (21.3)	84,224 (18.9)	13,292 (19.3)	16,973 (18.2)	12,258 (27.6)
Diagnostic period													
1980–89	157,443 (24.2)	116,766 (25.5)	29,073 (20.5)	11,604 (22.8)	24,896 (21.6)	28,523 (24.2)	33,320 (25.6)	36,070 (25.2)	34,634 (24.0)	115,238 (25.9)	19,539 (28.3)	17,418 (18.7)	5248 (11.8)
1990–99	229,566 (35.3)	162,827 (35.5)	49,447 (34.9)	17,292 (33.9)	41,457 (35.9)	40,943 (34.7)	45,402 (34.9)	50,154 (35.0)	51,610 (35.7)	159,513 (35.9)	24,196 (35.1)	32,311 (34.7)	13,546 (30.5)
2000–08	264,236 (40.6)	178,862 (39.0)	63,311 (44.6)	22,063 (43.3)	48,999 (42.5)	48,556 (41.1)	51,389 (39.5)	57,002 (39.8)	58,290 (40.3)	170,128 (38.2)	25,209 (36.6)	43,313 (46.6)	25,586 (57.7)
Status													
Alive	271,757 (41.7)	189,946 (41.4)	61,046 (43.0)	20,765 (40.8)	58,419 (50.6)	53,105 (45.0)	52,976 (40.7)	54,900 (38.3)	52,357 (36.2)	168,015 (37.8)	23,787 (34.5)	41,228 (44.3)	38,727 (87.3)
Died (cancer)^c^	259,616 (39.9)	183,671 (40.1)	54,966 (38.8)	20,979 (41.2)	38,846 (33.7)	44,072 (37.3)	52,338 (40.2)	60,307 (42.1)	64,053 (44.3)	186,802 (42.0)	31,730 (46.0)	38,703 (41.6)	2381 (5.4)
Died (other cause)	119,872 (18.4)	84,838 (18.5)	25,819 (18.2)	9215 (18.1)	18,087 (15.7)	20,845 (17.7)	24,797 (19.1)	28,019 (19.6)	28,124 (19.5)	90,062 (20.2)	13,427 (19.5)	13,111 (14.1)	3272 (7.4)

*SEIFA* Socio-Economic Index for Areas (1: least disadvantaged – 5: most disadvantaged quintile); Country of birth categorised into Australia, other English-speaking and non-English speaking countries and unknown

^a^Age at diagnosis missing for 48 cases throughout 1980–2008. ^b^Column percentages (due to rounding all percentages do not add up to 100%). ^c^Died due to the cancer of diagnosis

### Overall survival analyses

After adjusting for age, sex, diagnostic period, summary stage, cancer site and other study variables, people living in outer regional/remote areas had a slightly increased risk of cancer death compared with people living in major cities (SHR 1.05, 95% CI 1.03–1.06) (Model 3, Table 2). Compared with cases living in the least socio-economically disadvantaged areas, those living in all other SEIFA quintiles had an increased risk of cancer death, with the highest risk elevation observed for the most disadvantaged areas (SHR 1.15, 95% CI 1.13–1.17). People born in other English speaking countries had a similar risk (SHR 0.99, 95% CI 0.98–1.01), and people born in non-English speaking countries had a slightly lower risk of cancer death than the Australian-born (SHR 0.91, 95% CI 0.90–0.92). Results were similar after excluding cases with unknown summary stage (data not shown).

**Table 2:** Competing risk regression models of risk of cancer death, by remoteness, socio-economic disadvantage and country of birth category, NSW 1980–2008

	Unadjusted	Model 1^a^	Model 2^b^	Model 3^b^
Remoteness	SHR (95% CI)	SHR (95% CI)	SHR (95% CI)	SHR (95% CI)
Major cities	1	1	1	1
Inner regional	0.98 (0.97–0.99)	0.97 (0.96–0.98)	1.01 (1.00–1.02)	1.01 (1.00–1.02)
Outer regional/ Remote	1.06 (1.04–1.07)	1.02 (1.01–1.04)	1.04 (1.03–1.06)	1.05 (1.03–1.06)
SEIFA quintile				
1 (least disadvantaged)	1	1	1	1
2	1.14 (1.13–1.16)	1.11 (1.10–1.13)	1.05 (1.04–1.07)	1.04 (1.02–1.06)
3	1.26 (1.24–1.28)	1.19 (1.18–1.21)	1.09 (1.07–1.10)	1.08 (1.06–1.09)
4	1.35 (1.33–1.36)	1.26 (1.25–1.28)	1.12 (1.11–1.14)	1.11 (1.09–1.12)
5 (most disadvantaged)	1.46 (1.44–1.47)	1.37 (1.35–1.39)	1.17 (1.16–1.19)	1.15 (1.13–1.17)
Country of birth				
Australia	1	1	1	1
Other English speaking	1.14 (1.12–1.15)	1.10 (1.08–1.10)	1.00 (1.00–1.02)	0.99 (0.98–1.01)
Non-English speaking	1.01 (1.00–1.03)	1.09 (1.08–1.10)	0.93 (0.92–0.94)	0.91 (0.90–0.92)
Unknown	0.11 (0.11–0.11)	0.14 (0.13–0.14)	0.20 (0.19–0.21)	0.23 (0.22–0.24)

*SEIFA* Socio-Economic Index for Areas, *SHR* Sub-hazard ratio, *CI* Confidence interval; Country of birth categorised into Australia, other English-speaking and non-English speaking countries and unknown

^a^Model 1 adjusted for sex, age, diagnostic period and, as appropriate, for remoteness/SEIFA quintile/country of birth

^b^Model 2 further adjusted for cancer site and Model 3 further adjusted for summary stage

Interactions between diagnostic year and study variables indicated significant time variations in relation to socio-economic disadvantage quintiles (*p* < 0.001) but not in relation to remoteness (*p* = 0.511) or country of birth (*p* = 0.078) when unknown country of birth was excluded.

**Fig. 1:**
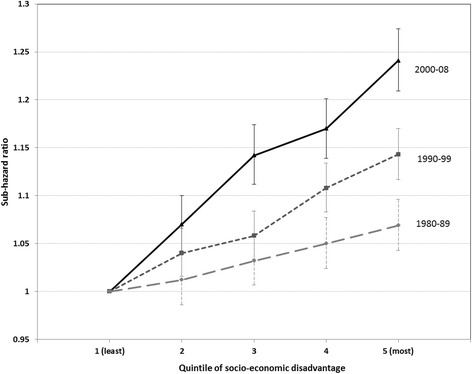
Sub-hazard ratios for socio-economic disadvantage quintiles 2–5 compared with quintile 1 for cases diagnosed in 1980–1989, 1990–1999 and 2000–2008. Sub-hazard ratios with 95% confidence intervals. Socio-economic disadvantage measured as the Index of Relative Socio-Economic Disadvantage and categorised into quintiles (1: least disadvantaged – 5: most disadvantaged). Models adjusted for sex, age, remoteness, country of birth, cancer site, and summary stage

Period-stratified analyses indicated largely constant trends over time in risk of cancer death by remoteness and country of birth (Table 3). However, when comparing the most with the least socio-economically disadvantaged areas, disparities in risk of death from cancer increased over time (SHR 1.07, 95% CI 1.04–1.10 for 1980–1989; SHR 1.14, 95% CI 1.12–1.17 for 1990–1999; and SHR 1.24, 95% CI 1.21–1.27 for 2000–2008) (Table 3, Fig. 1).

**Table 3:** Competing risk regression models of risk of cancer death by remoteness, socio-economic disadvantage and country of birth category, stratified by diagnostic period (1980–1989, 1990–1999, 2000–2008), NSW 1980–2008

	1980–1989 *(n = 157,400)*		1990–1999 *(n = 229, 561)*		2000–2008 *(n = 264,236)*	
	Site-adjusted^a^	Stage-adjusted^b^	Site-adjusted^a^	Stage-adjusted^b^	Site-adjusted^a^	Stage-adjusted^b^
Remoteness	SHR (95% CI)	SHR (95% CI)	SHR (95% CI)	SHR (95% CI)	SHR (95% CI)	SHR (95% CI)
Major cities	1	1	1	1	1	1
Inner regional	0.99 (0.97–1.01)	1.00 (0.98–1.02)	1.01 (0.99–1.03)	1.02 (1.00–1.04)	1.01 (0.99–1.03)	1.02 (1.00–1.04)
Outer regional/ Remote	1.03 (1.00–1.06)	1.04 (1.01–1.07)	1.06 (1.03–1.09)	1.07 (1.04–1.09)	1.03 (1.01–1.06)	1.03 (1.00–1.06)
SEIFA quintile						
1 (least disadvantaged)	1	1	1	1	1	1
2	1.01 (0.99–1.04)	1.01 (0.99–1.04)	1.06 (1.04–1.09)	1.04 (1.02–1.07)	1.07 (1.04–1.10)	1.07 (1.04–1.10)
3	1.04 (1.02–1.07)	1.03 (1.01–1.06)	1.08 (1.05–1.10)	1.06 (1.03–1.08)	1.15 (1.12–1.18)	1.14 (1.11–1.17)
4	1.06 (1.04–1.09)	1.05 (1.02–1.08)	1.13 (1.11–1.16)	1.11 (1.08–1.13)	1.17 (1.14–1.20)	1.17 (1.14–1.20)
5 (most disadvantaged)	1.08 (1.06–1.11)	1.07 (1.04–1.10)	1.17 (1.15–1.20)	1.14 (1.12–1.17)	1.25 (1.22–1.28)	1.24 (1.21–1.27)
Country of birth						
Australia	1	1	1	1	1	1
Other English speaking	0.99 (0.97–1.01)	0.99 (0.97–1.01)	1.01 (0.99–1.03)	1.00 (0.98–1.02)	1.02 (1.00–1.05)	1.00 (0.98–1.03)
Non-English speaking	0.91 (0.89–0.94)	0.91 (0.88–0.93)	0.93 (0.92–0.95)	0.92 (0.90–0.93)	0.93 (0.91–0.95)	0.90 (0.88–0.92)
Unknown	0.37 (0.35–0.40)	0.40 (0.37–0.43)	0.16 (0.15–0.18)	0.19 (0.17–0.20)	0.17 (0.15–0.18)	0.19 (0.17–0.20)

*SEIFA* Socio-Economic Index for Areas, *SHR* Sub-hazard ratio, *CI* Confidence interval; Country of birth categorised into Australia, other English-speaking and non-English speaking countries and unknown

^a^Models adjusted for sex, age, cancer site and, as appropriate, for remoteness/SEIFA quintile/country of birth

^b^Models further adjusted for summary stage

### Cancer site-specific survival analyses

Compared with major cities, living in outer regional/remote areas was generally associated with a slightly increased risk of cancer death, with the highest elevations observed for stomach (SHR 1.15, 95% CI 1.07–1.24), colon/rectum (SHR 1.12, 95% CI 1.08–1.17), cervix (SHR 1.24, 95% CI 1.07–1.43) and prostate cancers (SHR 1.10, 95% CI 1.05–1.16) (Table 4).

**Table 4:** Cancer site-specific competing risk regression models of risk of cancer death by remoteness, socio-economic disadvantage and country of birth category, NSW 1980–2008

	Stomach	Colorectal	Liver	Lung	Melanoma	Breast^a^	Cervix	Prostate
	*(n = 17,848)*	*(n = 101,402)*	*(n = 5732)*	*(n = 72,437)*	*(n = 70,464)*	*(n = 88,768)*	*(n = 8968)*	*(n = 95,543)*
Remoteness	SHR (95% CI)^b^	SHR (95% CI)^b^	SHR (95% CI)^b^	SHR (95% CI)^b^	SHR (95% CI)^b^	SHR (95% CI)^b^	SHR (95% CI)^b^	SHR (95% CI)^b^
Major cities	1	1	1	1	1	1	1	1
Inner regional	1.09 (1.04–1.14)	1.02 (0.99–1.05)	1.04 (0.95–1.13)	1.02 (0.99–1.04)	0.97 (0.92–1.03)	1.00 (0.96–1.04)	1.04 (0.93–1.16)	1.08 (1.05–1.12)
Outer regional/Remote	1.15 (1.07–1.24)	1.12 (1.08–1.17)	1.08 (0.95–1.24)	1.02 (0.99–1.06)	1.09 (1.00–1.19)	1.04 (0.98–1.10)	1.24 (1.07–1.43)	1.10 (1.05–1.16)
SEIFA quintile								
1 (least disadvantaged)	1	1	1	1	1	1	1	1
2	0.99 (0.93–1.05)	1.05 (1.01–1.08)	1.01 (0.90–1.12)	1.06 (1.02–1.09)	1.10 (1.02–1.18)	1.06 (1.01–1.11)	0.94 (0.80–1.10)	1.06 (1.01–1.11)
3	1.01 (0.95–1.07)	1.06 (1.02–1.10)	1.07 (0.96–1.18)	1.08 (1.05–1.12)	1.13 (1.05–1.22)	1.12 (1.07–1.18)	1.10 (0.95–1.27)	1.08 (1.03–1.13)
4	1.03 (0.97–1.09)	1.11 (1.07–1.14)	1.11 (1.00–1.23)	1.12 (1.09–1.15)	1.12 (1.04–1.21)	1.14 (1.09–1.19)	1.13 (0.98–1.30)	1.13 (1.08–1.19)
5 (most disadvantaged)	1.03 (0.97–1.09)	1.14 (1.10–1.18)	1.22 (1.11–1.34)	1.15 (1.12–1.18)	1.25 (1.16–1.34)	1.14 (1.08–1.19)	1.11 (0.97–1.27)	1.16 (1.11–1.22)
Country of birth								
Australia	1	1	1	1	1	1	1	1
Other English speaking	1.00 (0.95–1.06)	0.97 (0.93–1.00)	0.89 (0.80–1.00)	0.98 (0.95–1.00)	0.94 (0.86–1.03)	1.00 (0.95–1.04)	0.83 (0.73–0.94)	1.03 (0.99–1.08)
Non-English speaking	0.87 (0.83–0.91)	0.92 (0.89–0.95)	0.84 (0.78–0.90)	0.88 (0.86–0.90)	1.26 (1.14–1.39)	0.98 (0.94–1.02)	0.82 (0.73–0.92)	0.93 (0.89–0.97)
Unknown	0.49 (0.41–0.58)	0.32 (0.28–0.36)	0.35 (0.25–0.50)	0.48 (0.43–0.53)	0.03 (0.03–0.04)	0.27 (0.23–0.32)	0.14 (0.09–0.22)	0.13 (0.11–0.15)

*SEIFA* Socio-Economic Index for Areas, *SHR* Sub-hazard ratio, *CI* Confidence interval; Country of birth categorised into Australia, other English-speaking and non-English speaking countries and unknown

^a^Female breast cancers only

^b^Models adjusted for sex (when appropriate), age, diagnostic period, summary stage and, as appropriate, for remoteness/SEIFA quintile/country of birth

Living in the most socio-economically disadvantaged areas compared with the least disadvantaged areas was generally associated with higher risk of cancer death, with the most pronounced disparities observed for liver cancer (SHR 1.22, 95% CI 1.11–1.34) and melanoma (SHR 1.25, 95% CI 1.16–1.34); followed by prostate (SHR 1.16, 95% CI 1.11–1.22), lung (SHR 1.15, 95% CI 1.12–1.18), colon/rectum (SHR 1.14, 95% CI 1.10–1.18), and breast cancer (SHR 1.14, 95% CI 1.08–1.19).

People born in non-English speaking countries generally had a lower risk of cancer death than the Australian-born, with the exception of melanoma (SHR 1.26, 95% CI 1.14–1.39) and breast cancer (SHR 0.98, 95% CI 0.94–1.02, i.e., no statistically significant difference). People born in other English speaking countries had similar risks of cancer death to the Australian-born, with the exception of a lower risk for cervix cancer (SHR 0.83, 95% CI 0.73–0.94).

Results remained largely similar when cases with unknown summary stage were excluded, with the following exceptions:

- Liver cancer
  - SHR 0.95, 95% CI 0.85–1.07 for people living in inner regional areas
  - SHR 0.83, 95% CI 0.72–0.96 for people born in other English speaking countries
- Breast cancer
  - SHR 0.93, 95% CI 0.89–0.97 for people born in non-English speaking countries

Results remained unchanged when excluding those with unknown country of birth (not shown).

All final competing risk regression models were found to satisfy proportional hazards assumptions.

## Discussion

After accounting for differences in cancer site, summary stage at diagnosis and death from competing events, people diagnosed with cancer and living in socio-economically disadvantaged areas had an overall elevated risk of cancer death, with an increasing comparative risk of cancer death over time with increasing socio-economic disadvantage. Elevated risk of cancer death was also detected for people with cancer living in outer regional/remote areas whereas overseas-born people with cancer had similar/lower risk of cancer death than Australian-born. Although effect sizes were generally small, they point to possible areas of disadvantage probably including pockets of more severe disadvantage than applying to these areas overall. As in previous studies reviewed by Quaglia et al. [6], we found socio-economic cancer survival disparities to remain after adjusting for summary stage. Similarly, socio-economic survival disparities remained, although diminished, after adjustment for cancer site illustrating that differences in cancer mix cannot entirely explain disparities. In addition to potential residual confounding, factors likely to have contributed to remaining disparities include differences in health behaviours, co-morbidities, social support, access to and quality of treatment and screening services, and processes for clinical follow-up [1, 6].

Our findings are consistent with the previous international and Australian literature about cancer survival disparities [3, 6, 7, 10, 16, 34] and show that these disparities exist after accounting for competing risk events. Our results are similar to those of studies using cause-specific survival, which may reflect independence of competing causes of death [20]. For bias to arise in cause-specific modelling, competing risks have to be common compared with the outcome of interest and the counterfactual risk of the outcome of interest must differ between those censored and those not censored due to competing events [35].

In site-specific analyses, we found the strongest socio-economic survival gradients for liver, melanoma, prostate, lung, colon/rectum, and breast cancer. Previous Australian studies have reported generally similar findings [7, 10]. Notably, we found a socio-economic survival gradient for melanoma but not for stomach cancer in contrast to a recent study from NSW [10]. We used CD-based SEIFA measures, which may have been more sensitive in detecting a melanoma survival gradient. Accounting for competing risks may partly explain the differences in stomach cancer. Internationally, highest relative risks have been reported for intermediate or good prognosis cancers [6]. There may be less potential for socio-economic factors to impact cancers with overall poor prognosis. Our findings in relation to socio-economic survival gradients for liver and lung cancers may be explained by differences in risk behaviour and co-morbidities.

We observed poorer cancer survival with increasing remoteness for stomach, colon/rectum, cervix and prostate cancers. For colon/rectum, cervix and prostate cancers, these findings may partly be explained by access to and uptake of screening services and other early-detection initiatives. However, in recent years rural cancer disparities have been actively addressed by establishing regional cancer centres and adopting novel approaches, such as telehealth, to improve rural cancer services in Australia [36]. In addition, differentiating the interconnected effects of remoteness and socio-economic disadvantage can be challenging with existing aggregate measures [8].

After adjusting for cancer site and summary stage, overseas-born people diagnosed with cancer in Australia generally had similar/lower risk of cancer death than Australian-born. The extent to which this is artefactual reflecting loss to follow-up of cases returning to their country of origin, or a healthy-migrant effect (i.e., migrants are commonly healthier than general population [37]), is not known. There is evidence from the United States that findings of cancer survival advantage among Hispanics and Asians may be biased due to incomplete follow-up and missing death information [38]. Under-notification of country of birth for cancers with a good prognosis (such as melanoma) is likely to at least partly explain the relatively low risk of death among people with unknown country of birth. The NSW CR receives country of birth information mostly through death registrations and hospital admission notifications. If the registry only receives a pathology notification, country of birth will be missing. Excluding those with unknown country of birth in a sensitivity analysis had no impact on results.

When considering the results of both interaction and period-specific analyses, an increasing comparative risk of death from cancer was observed over time by socio-economic disadvantage but not by remoteness or country of birth. Stanbury et al. reported that socio-economic cancer disparities for several cancer sites (stomach, colorectal, liver, lung, breast and prostate) remained in NSW over 1996–2008 [10]. Studies from the UK and New Zealand have shown that improvements in cancer survival over time are likely to be slower for more deprived population groups due to several reasons, including poorer access to improved treatments, co-morbidities and stage at diagnosis [13, 14]. Another UK study highlighted the importance of healthcare related factors on changes in cancer survival disparities over time [39]. New interventions may be less accessible to deprived populations leading to increased inequalities [40]. Further research is needed on the extent that this applies to Australia.

### Study limitations

We showed the hazard of cancer death by socio-economic disadvantage to increase over time in relative terms. Results based on area-level aggregate measurements are likely to be biased toward the null and potentially underestimate the real extent of survival inequalities, especially when deprivation measures are based on large geographic areas [41]. Therefore, we used socio-economic disadvantage measure based on the smallest geographic units available (CDs). Changes in SEIFA measures over time may also have impacted our results. However, we conducted comparative analyses using equal-population quintiles and, therefore, this impact is likely to be minor. The remoteness measure used in this study for 1980–1999 was based on 2006 SLAs and may not be representative of the previous 20+ years, which may partly explain why we did not find changes in survival over time by remoteness.

There is likely to be stage migration over 1980–2008, but we do not believe that this would have caused a markedly different impact across the different study groups. In addition, models were adjusted/stratified by diagnostic period and stage. We did not have information on treatments or co-morbidities which are likely to influence cancer survival [6]. We were not able to examine differences in survival for 1980–2008 by Aboriginal and Torres Strait Islander status, due to under-recording of Aboriginal status in health registries especially prior to the late 1990s. Countries of birth were categorised using broad categories of other English and non-English speaking countries. Bias due to misclassification of cause of death being potentially associated with study variables [35] cannot be completely ruled out.

## Conclusions

Active attention is needed to address cancer survival disparities by socio-economic disadvantage, especially as these disparities appear to have increased. Reasons behind these disparities should be further examined in order to plan targeted actions. Collaboration between different stakeholders, including policy makers, government and health care providers, is important to ensure comprehensive approach as disparities are likely to be driven by multiple, complex processes. A policy emphasis on socio-economic disadvantage is required if the trend of the widening gap in death from cancer by socio-economic disadvantage is to be reversed.

## Abbreviations

ABS: Australian Bureau of Statistics
ARIA: Accessibility/Remoteness Index of Australia
CD: Census Collection District
CI: Confidence interval
DCO: Death certificate only cases
ICD: International Classification of Diseases
IRSD: Index of Relative Socio-Economic Disadvantage
NDI: National Death Index
NSW CR: New South Wales Cancer Registry
NSW: New South Wales
RA: Remoteness area
SEIFA: Socio-Economic Index for Areas
SHR: Sub-hazard ratio
SLA: Statistical Local Area
UK: United Kingdom
